# The lymphatic system as a potential mechanism of spread of melioidosis following ingestion of *Burkholderia pseudomallei*

**DOI:** 10.1371/journal.pntd.0009016

**Published:** 2021-02-22

**Authors:** Michelle Nelson, Alejandro Nunez, Sarah A. Ngugi, Timothy P. Atkins

**Affiliations:** 1 CBR Division, Defence Science and Technology Laboratory (Dstl), Porton Down, Salisbury, Wiltshire, United Kingdom; 2 Animal and Plant Health Agency, Weybridge, Addlestone, Surrey, United Kingdom; University of Texas Medical Branch, UNITED STATES

## Abstract

*Burkholderia pseudomallei* is the causative agent of melioidosis, which is a Gram negative, facultative intracellular bacterium. Disease is prevalent in SE Asia and in northern Australia, as well as in other tropical and subtropical regions. Recently, there is an increasing awareness of the importance of bacterial ingestion as a potential route of infection, particularly in cases of unexplained origin of the disease. The marmoset is a New World Monkey (NWM) species that is being developed as an alternative NHP model to complement the more traditionally used Old World Monkeys (OWM). Models have been developed for the traditional routes of disease acquisition, subcutaneous and inhalational. This manuscript details the development and characterisation of an ingestion model of melioidosis. Dose-ranging study assessed the lethality of *B*. *pseudomallei* and disease progression was assessed by euthanizing animals at predetermined time points, 12, 36, 48 and 54 hours post-challenge. Challenge doses of greater than 6.2 x 10^6^ cfu resulted in an acute, lethal, febrile disease. Following challenge the lung was the first organ, outside of the gastrointestinal tract, to become colonised. Enteritis (duodenitis, ileitis and/or jejunitis) was observed in sections of the small intestine from animals that succumbed to disease. However, the most severe pathological features were observed in the mesenteric lymph nodes from these animals. These findings are consistent with lymphatic draining as route of dissemination.

## Author summary

*Burkholderia pseudomallei* is a bacteria that causes the disease melioidosis. Disease is usually caused by bacteria entering cuts in the skin or by breathing in the bacteria. But disease can also be caused by drinking water containing the bacteria, but this form of the disease is not well understand. Other researchers have looked at what happens when mice are given the bacteria via the mouth. This manuscripts looks at what happens when small monkeys, marmoset drink the bacteria. Different amounts of bacteria were given to the marmosets to determine how many bacteria are needed to cause disease. More than 6 million bacteria caused animals to have a fever and become sick enough to be euthanized. The bacteria travelled from the stomach to the intestine where there were small areas of swelling with lots of immune cells than caused damage to the tissue. The bacteria then went to the lungs really early in the disease and then spread to other parts of the body.

## Introduction

*Burkholderia pseudomallei* is the causative agent of melioidosis, which is a Gram negative, facultative intracellular bacterium that is classified as a as HHS/CDC Tier 1 agents (7 CFR Part 331, 9 CFR Part 121, and 42 CFR Part 73). Disease is prevalent in SE Asia and in northern Australia but is increasingly becoming a major cause of morbidity and mortality in other tropical and subtropical countries [1]. The disease presents with diverse clinical manifestations, varying from acute sepsis, through chronic localized pathology, to latent infection. The mortality rate in acute infection is high, between 10 and 50% [2]. Disease presentation and mortality is believed to be associated with a number of parameters including bacterial strain, inoculation dose, route of entry and host factors [3,4]. Naturally occurring infection was primarily considered to be through bacterial entry via cuts or skin abrasions or via inhalation of infected soil or water particles [4]. Recently, there is an increasing awareness of the importance of bacterial ingestion as a potential route of infection, particularly in cases of unexplained origin of the disease. Indeed, *B*. *pseudomallei* has been isolated from drinking water in both Thailand and Northern Australia [5]. There is a high correlation between presence of bacteria in the drinking water and acquisition of melioidosis by ingestion [6]. Ingestion of the bacteria is suggested to be the cause of the incidence of melioidosis-related parotitis in children in Thailand, and possibly other parts of south east Asia, that is not observed in Australia [7,8]. Incidence of ingestion in animals is also highly suspected, and parotid infection has been observed in pigs that drank water from infected bore holes [9].

Oral infection has been demonstrated in mice leading to either an acute, partially lethal disease with challenge doses of between ~10^6^ and 10^8^ cfu, or a more chronic, persistent disease with challenge doses of between ~10^2^ and 10^5^ cfu [10,11]. No difference in survival was observed with the two inbred mouse species used, C57Bl/6 and Balb/c. The former having a Th1 biased immune response and the latter a Th2 biased immune response. In these studies, disease pathogenesis and dissemination was distinctly different from other characterized routes of infection, specifically with greater colonization in the gut. The impact of the different disease presentation on the efficacy of medical countermeasures has not been explored. There are no licensed human vaccines for melioidosis and antibiotic treatment is often prolonged, complex and requires intravenous administration. Hence, there is a need to develop reliable, effective medical countermeasures. Understanding the impact that the route of acquisition has on the disease presentation is vital to the development of medical countermeasures.

The common marmoset (*Callithrix jacchus*) is a New World Monkey (NWM) species that is being developed as an alternative NHP model to complement the more traditionally used Old World Monkeys (OWM) (e.g. rhesus and cynomolgus macaques). The marmoset provides a technically viable and alternative for pre-clinical modelling of human infectious diseases [12]. The basic biology of marmosets is similar to that of humans and commercial reagents (primarily of human origin) are available for immunological assays of responses to infection. Additionally, there are numerous advantages to using marmosets; their smaller size (300 to 500 g) means it is easier to house larger numbers under biosafety level (BSL) 3 restrictions with a variety of environmental stimuli. For studying ingestional disease, the anatomy of the marmoset’s gastrointestinal tract is more similar to humans than mice. Mice have a non-glandular fore stomach and a smooth mucosa layer in the small intestine unlike both humans and marmosets [13,14,15]. The fore-stomach in mice is associated with food storage and may be the reason that high bacterial colonisation was observed with oral delivery of *B*. *pseudomallei* [10,11].

The aim of these studies was to develop and characterise an ingestion model of melioidosis to facilitate direct comparison of the disease in a single animal species closely related to humans.

## Materials and methods

### Ethics statement

All animal studies were carried out in accordance with the UK Animals (Scientific Procedures) Act of 1986 and the Codes of Practice for the Housing and Care of Animals used in Scientific Procedures 1989. The work was performed under a Project licence (P18072309) that was approved by the UK Home Office and Animal in Sciences Committee (an independent committee that offers advice to The Secretary of State of the ethics of the proposed work).

### Animals

Healthy, sexually mature common marmosets (*C*. *jacchus*) were obtained from the Dstl Porton Down breeding colony and housed in vasectomised male and female pairs. The mean age and weight of the animals used 3.2 ± 1.4 years and 436 ± 50g at the time of challenge. All animals were allowed free access to food and water as well as environmental enrichment. All animals were surgically implanted intraperitoneally with a Remo 200 device (Remo Technologies Ltd, Salisbury, UK) under general anaesthesia (Ketamine/Domitor/Isofluorane) to record core body temperature (Tc). Prophylactic pain relief of 0.2 mg/Kg of meloxicam and 0.005 mg/Kg of buprenorphine was administered. Data were transmitted from the devices at 30 second intervals to locally placed antennas and relayed to receivers. Data were analysed using the eDacq software to provide real-time and recordable Tc (EMMS, Bordon, Hampshire, UK). At least 7 days prior to challenge, baseline blood was collected from all animals to assess baseline levels of immunology, clinical chemistry and haematology parameters. Animals were anesthetised with 5 mg of ketamine hydrochloride and a 23-gauge needle was attached to a 5 mL syringe and up to 2.5 mL of blood was collected from the femoral vein.

Marmosets were challenged orally via a syringe containing 100 μL of *B*. *pseudomallei* and 1 mL of Nesquik milkshake powder mixed with water. Animals had previously been conditioned to accept the uninfected solution from the syringe, and in all cases accepted the infected solution readily and drank with no evidence of aspiration. Following challenge, animals were observed at least three times per day after challenge, for up to 18 days post-challenge. Clinical signs were scored during physical entry into the room and temperature and remote camera observations were recorded during silent hours. Animals were euthanised based on a 2°C temperature drop in conjunction with evidence of at least 3 clinical signs. The temperature drop of 2°C is based on normal core body temperature of the animals at that time of day (e.g. 34°C during the night or 36°C during the day), that is determined for at least three days pre-study. Animals were euthanized with 2 to 3 mL of pentobarbitone administered intra-peritoneally, following sedation with 10 mg of ketamine hydrochloride.

Following challenge with *B*. *pseudomallei*, animals were handled under animal containment level 3 (CL3) conditions, within a half-suit isolator compliant with British Standard BS5726.

In order to determine the lethality of *B*. *pseudomallei* by the ingested route and assess the disease progression, two studies were performed; a dose-ranging study (Study 1) and a natural history study (Study 2). The dose ranging study was performed in a step-wise manner using pairs of animals at each step using a modified Dixon staircase method [16]. The initial challenge dose (2.7 x 10^8^ cfu) was selected based on the highest achievable dose. Following confirmation of lethality, the second challenge dose (1.3 x 10^4^ cfu) was selected as this dose that is lethal in marmosets by both the inhalational and subcutaneous routes [17,18]. Subsequent challenge doses were selected based on the outcome of the previous step; a high dose was selected if the animals survived, a lower dose selected if both animals succumbed.

In the second study, marmosets were challenged in two cohorts (A and B) with either 7.2 x 10^6^ or 8.1 x 10^6^ cfu of *B*. *pseudomallei* by the ingested route. One pair of animals from each cohort was euthanized at predetermined time points, 12, 36, 48 and 54 hours post-challenge to assess disease progression.

### Bacterial strain and culture

Glycerol stocks of *B*. *pseudomallei* strain K96243 were provided by Battelle Biomedical Research Centre. A frozen stock of *B*. *pseudomallei* was thawed and streaked onto Luria Bertani supplemented with 5% glycerol (LBG) agar plates. The plates were incubated at 37°C for between 23.5 and 25.3 hours (mean time of 23.9 ± 0.27 hours). A loopful of the colonies were used to inoculate approximately 5 mL of PBS and the optical density (OD_600_) determined (CO7500 Colorimeter, WPA Colourwave). The OD_600_ was adjusted to between 0.35 to 0.38, which is equivalent to approximately 1 x 10^8^ cfu/mL. One mL of final suspension was used to inoculate 100 mL LB broth. The broth cultures were incubated at 37°C on a rotary shaker (Innova 4200 Shaking Incubator, New Brunswick Scientific) at 180 rpm for a between 15 and 16.9 hours (mean time of 16.3 ± 0.27 hours). The broth was removed from the shaker and the OD_600_ measured in triplicate using 1 mL aliquots in a cuvette. The OD_600_ of 1 mL of the 100 mL culture was adjusted with PBS to between 0.35 to 0.38, which is equivalent to approximately 1 x 10^8^ cfu/mL. A series of ten-fold dilutions (10^−1^ to 10^−8^) was prepared by aliquotting 1 mL of the OD adjusted culture into 9 mL of PBS. The appropriate dilution was selected for challenge. In parallel, the dilution series was used to determine the viable count by aliquotting a single 250 μL aliquot of each of the 10^−5^, 10^−6^, 10^−7^, and 10^−8^ dilution onto four separate LBG agar plates. Plates were incubated at 37°C for between 24 and 48 hours prior to enumeration.

### Post-mortem analysis

Post-mortem examinations were performed on all animals in all studies; organs removed were assessed for bacteriology, macroscopic and microscopic features, and immunohistochemical analysis. Blood was removed from terminally anaesthetised marmosets by cardiac puncture for assessment of bacteraemia, immunology, clinical chemistry and haematological parameters.

### Bacteriology

Bacterial loads were determined in blood, liver, spleen, kidneys, and lungs. Organs were removed aseptically and processed as previously described [19]. A series of ten-fold dilutions (10^−1^ to 10^−8^) was prepared using 0.1 mL aliquots and 0.25 mL of the neat to 10^−8^ dilutions were sub-cultured, in duplicate, onto LB-agar plates for *B*. *pseudomallei* and incubated at 37°C for 24 h. Counts are expressed as cfu.g^-1^ of tissue or cfu.mL^-1^ of blood.

### Immunological

Single cell suspensions of blood (collected pre-and post-challenge), lung and spleen were stained using stain sets to identify cell markers and the cell phenotype and activation status by flow cytometry (S1 Table). Stained cells were fixed for 48 hours in a final volume of 4% paraformaldehyde. Samples were analysed on a BD FACSCanto II and cell populations determined using BD FACSDiva software. Whole cells (as determined by the presence of an intact nucleus but not necessarily an intact cell membrane) were detected by nuclear staining allowing the area of interest to be defined by forward and side scatter. Forward and side scatter were also used to gate areas for detection of lymphocytes (T and B cells), natural killer cells (NK), macrophages (M0), and neutrophils.

### Histopathology

Tissues were fixed in 10% neutral buffered formalin and processed for paraffin wax embedding using standard techniques. Thin sections (4 μm) were cut and stained with haematoxylin and eosin (H&E) for histopathological analysis. A scoring system based on the type of lesions and organ distribution was established in order to allow semi-quantitative comparison [20].

### Immunohistochemistry

Immunohistochemical staining (IHC) was performed on selected tissues (liver, spleen, lungs, GI tract) for the detection of bacterial antigen, T-cells (CD3^+^), macrophages and neutrophils (MAC387^+^). A semi-quantitative scoring system was established in order to allow comparison [20]. The avidin biotin complex (ABC, Vector Elite; Vector laboratories) method was used for immunolabelling. Four μm sections of formalin-fixed wax-embedded tissues were prepared and dried onto polylysine slides (VWR Ltd). The sections were dewaxed, rehydrated and then treated in hydrogen peroxide 3% (v/v), in methanol for 15 minutes to eliminate endogenous peroxidase activity. The tissue sections were then pre-treated for antigen retrieval by either enzymatic digestion with pronase XIV (0.05% w/v pronase XIV 5.2U/mg; Sigma P5147-1G) and trypsin/alpha-chymotrypsin (0.5% trypsin and 0.5% alpha chymotrypsin; Sigma-Aldrich, Gillingham, Dorset, UK) at 37°C for 10 minutes. The sections were then microwaved in citric acid buffer (2.1g citric acid; Fisher Scientific, Loughborough, Leicestershire, U.K., in 1000 mL distilled water pH 6.0) for 18 minutes at 100°C, 90% effect (780W) or microwaved in Dako high pH 9.0 buffer for 10 minutes. The sections were then mounted in a Sequenza Immunostaining Centre (Shandon Scientific, Runcorn, UK) and rinsed with Tris buffered saline (TBS) pH 7.6, 0.005 M (Sigma–Aldrich, USA). Primary antibody cross-reactivity with tissue constituents was prevented using 1.5% normal serum block applied to the sections for 20 minutes. The serum block matched the host species in which the link antibody was raised. Details of primary antibodies used, specificity, concentration and incubation time are summarized in S1 Table. All primary antibodies had been previously screened to determine the optimum dilution and incubation temperature. The sections were washed in TBS and then incubated for 30 minutes with the appropriate biotinylated secondary link antibody (Vector Laboratories) before being washed twice in TBS again. The sections were incubated for 30 minutes at room temperature with Avidin Biotin complex (Vector Elite Kit, Vector Laboratories) and the signal was detected using 3,30-diaminobenzidine tetrahydrochloride (DAB). Finally the sections were lightly counterstained with Mayer’s haematoxylin (Surgipath, Peterborough, UK) for 5 minutes, dehydrated in absolute alcohol and cleared in xylene before being coverslipped.

IHC techniques for cell markers applied in this study have been successfully used in multiple species tissue samples (CD3^+^ and MAC387^+^ Bacterial capsular antigen was detected by using a *B*. *pseudomallei* antibody (3VIE5) that has shown cross reactivity with *B*. *mallei* [21]. Controls were included in each IHC run. These included sequential sections with an isotype control for each primary antibody, and the omission of the primary antibody.

### Haematology, clinical chemistry and electrolytes

Blood was collected from all animals at post-mortem into blood tubes, anti-coagulated with either EDTA or Lithium heparin. Key haematological parameters were measured from EDTA anti-coagulated blood by use of a laser-flow cytometry-based haematological analyser (ProCyte, IDEXX Laboratories Ltd, Bucks, UK). Plasma from heparinised blood was used to assess the clinical chemistry and electrolyte parameters were analysed using a ‘dry-slide’ technology biochemistry analyser (Catalyst Dx, IDEXX). Post-challenge data were compared to mean pre-challenge results.

### Statistics

Pearson’s correlation analysis was used to determine the relationship of gender, body weight, time to death and inhaled dose. Comparative analysis of bacteriology, immunology and blood chemistry data was performed using two-way ANOVA. Survival data was analyzed using a log rank (Mantel-Cox) test. Correlation of dose and time to euthansia was performed using Spearman Correlation.

## Results

### Lethality and disease progression

**Fig 1:**
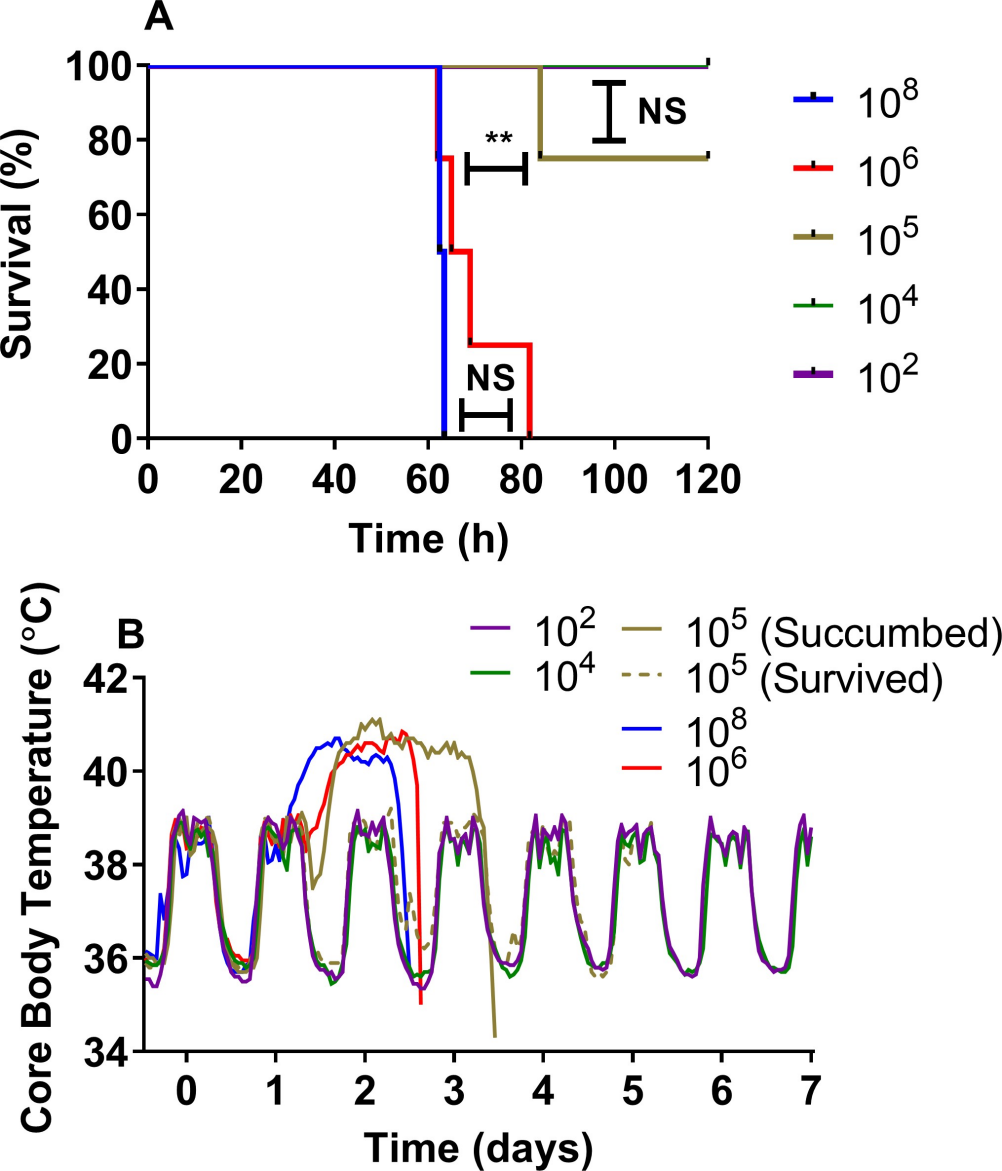
Observations in marmosets challenged with various doses of *B*. *pseudomallei* by the ingestion route of administration (Study 1; dose-ranging studies). A Survival plot, B Mean Core Body Temperature data. n = 2 for all groups of animals except the10^6^ group where 4 animals were used to determine reproducibility.

Initially, a dose-ranging study assessed the lethality with animals were challenged with between 1.3 x 10^2^ to 2.7 x 10^8^ cfu of bacteria by the ingested route (Table 1 and Fig 1A). In the second study, marmosets were challenged in two cohorts (A and B) with either 7.2 x 10^6^ or 8.1 x 10^6^ cfu of *B*. *pseudomallei* by the ingested route. One pair of animals from each cohort was euthanized at predetermined time points, 12, 36, 48 and 54 hours post-challenge to assess disease progression.

**Table 1:** Dose, survival and fever of animals in the Dose ranging Studies.

Animal ID	Dose (cfu)	^1^Time to onset of hypothermia (hours)	^2^Time to onset of fever (hours)	Duration of fever (hours)
**Marmoset 1**	2.70 x 10^8^	61.7	32.8	28.9
**Marmoset 2**	2.70 x 10^8^	60.4	35.7	24.8
**Marmoset 3**	1.33 x 10^2^	n/a	NF	NF
**Marmoset 4**	1.33 x 10^2^	n/a	NF	NF
**Marmoset 5**	1.33 x 10^4^	n/a	NF	NF
**Marmoset 6**	1.33 x 10^4^	n/a	NF	NF
**Marmoset 7**	6.20 x 10^5^	81.4	40.5	40.9
**Marmoset 8**	6.20 x 10^5^	n/a	NF	NF
**Marmoset 9**	6.20 x 10^6^	68.9	42.0	26.9
**Marmoset 10**	6.20 x 10^6^	64.0	34.9	29.1
**Marmoset 11**	6.79 x 10^5^	n/a	NF	NF
**Marmoset 12**	6.79 x 10^5^	n/a	NF	NF
**Marmoset 13**	6.79 x 10^6^	81.6	46.3	35.3
**Marmoset 14**	6.79 x 10^6^	61.2	38.7	22.6

^1^ This time represents a temperature drop to 39°C, following fever, in order to standardise to facilitate comparison between animals.

^2^fever defined as a temperature of 40°C for at least 3 consequence readings n/a is not applicable as animals survived to the end of the study

NF is no fever observed

Regardless of the challenge dose, all animals maintained their normal diurnal rhythm for a mean time of 38.7 ± 4.7 hours post-challenge (p.c.), (range from 32 to 46 hours), prior to the onset of fever (defined as greater than 40°C for at least 3 consecutive readings). Only animals that went on to succumb to disease exhibited a febrile response which lasted for a mean time of 29.8 ± 6.4 hours (Fig 1B). Animals remained febrile for a further 22 to 41 hours, prior to a decline in temperature as the animals reached the humane endpoint. Clinical signs were first observed between 44 to 48 hours p.c. which initially presented as an unkempt coat, reluctance to move and a change in behaviour with the animals becoming more subdued. This progressed within 24 hours, when animals exhibited a hunched posture, a red face and occasionally sunken or partially closed eyes. Pronounced hunching, reluctance to move and severe reddening of the face preceded the humane endpoint. Animals challenged with less than 1.3 x 10^4^ cfu of bacteria survived the duration of the study, with no overt clinical signs or fever. Only 25% of animals challenged with between 6.2 x 10^5^ and 6.8 x 10^5^ cfu succumbed to disease. All animals challenged with greater than 6.2 x 10^6^ cfu of bacteria succumbed to disease between 60 and 81 hours post-challenge. There was a statistical correlation between the dose the animals received and the time to euthanasia (Spearman Correlation, R = 0.83, P <0.001). No febrile response or clinical signs were observed in any animal that survived until the end of the study.

### Bacteriology & physiological response following ingested challenge

**Fig 2:**
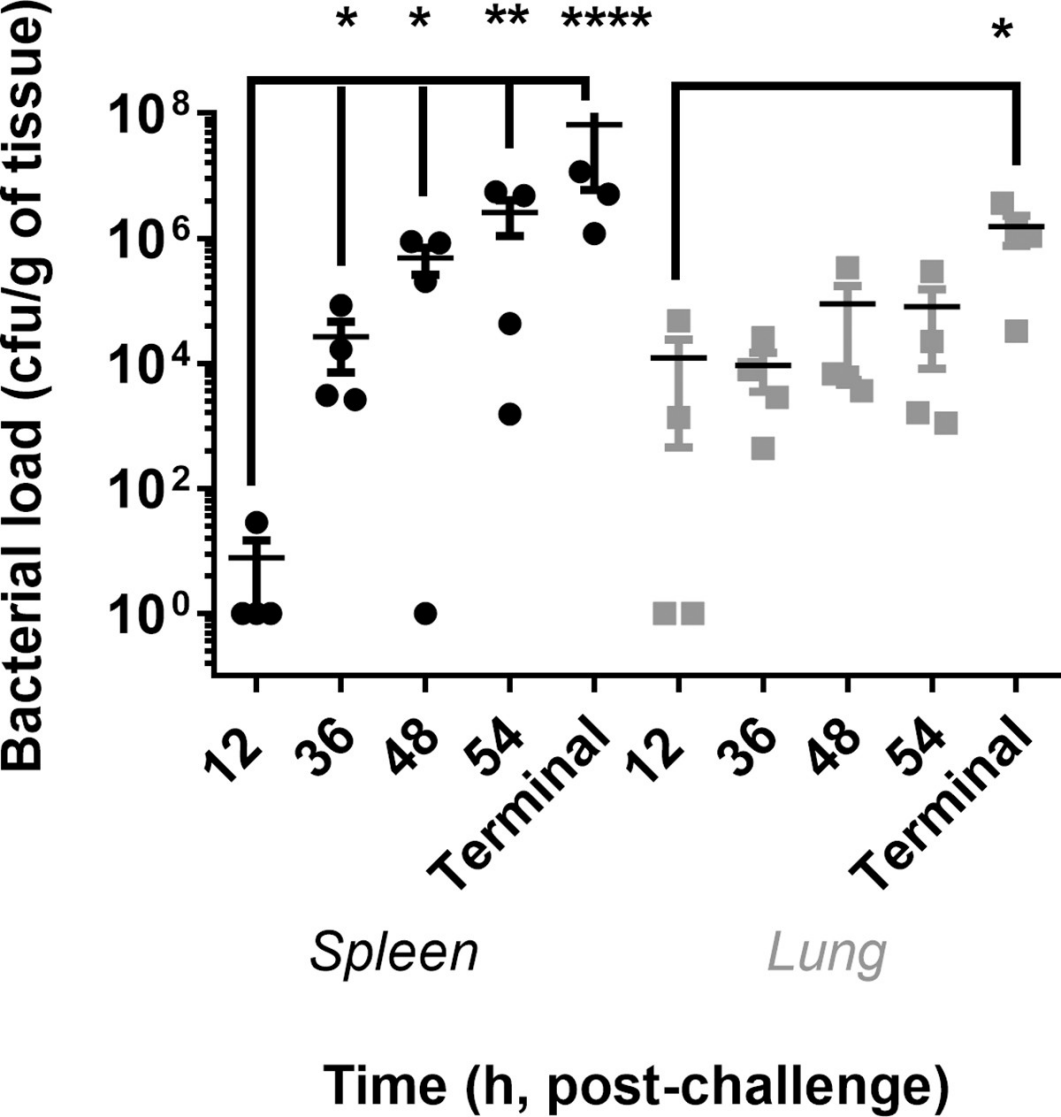
Bacterial load in the spleen and lungs of marmoset at post-mortem following challenge with *B*. *pseudomallei* by the ingestion route. All data is from Study 2, natural history study, except for the “Terminal” timepoint where data from animal’s challenged with between 6.2 and 6.8 x 10^6^ cfu of *B*. *pseudomallei* has been included for comparison.

At 12 hours p.c., the highest concentration of disseminated bacteria was evident in the lungs with up to 4.8 x 10^4^ cfu per g of tissue (Fig 2). Lower concentrations were observed in the kidney (up to 9.1 x 10^2^ cfu), liver (up to 6.5 x 10^2^ cfu) and the spleen (29 cfu in one animal only) (Figs 2 and S1). No bacteria were observed in the blood at this time (S1 Fig). From 36 hours p.c. the concentrations of bacteria in the spleen increased rapidly, with this organ having the highest bacterial load at the remaining time points. The next highest concentration of bacteria was observed in the lungs with levels remaining consistent at 48 and 54 hours p.c., increasing at the humane endpoint. Bacteraemia was only evident in two animals at 36 hours p.c., one animal at 48 hours p.c. and two animals at 54 hours p.c. In animals that succumbed to disease, the highest concentrations of bacteria were isolated from the liver and spleen of all animals, ranging from between 4.8 x 10^6^ to 4.6 x 10^9^ cfu in the liver and 1 x 10^6^ to 2.4 x 10^8^ cfu in the spleen. In the lungs and blood, levels of between 3.3 x 10^4^ to 3.3 x 10^7^ and 1.5 x 10^4^ to 6.8 x 10^5^ cfu were recovered, respectively. No bacteria were recovered from any of the animals that survived the duration of the study. Therefore, in all cases, the levels of bacteria recovered in animals that succumbed to disease were significantly different to the animals that survived.

The bacterial load was assessed in the gastrointestinal tract, initially by plate culture in animals that succumbed to disease (Study 1) and then using immunohistochemistry (IHC)(Study 2) (Table 2). The number of bacteria isolated from homogenised tissue from the gastrointestinal tract ranged from 1.2 x10^5^ to 2.9 x 10^7^ cfu/g in animals that succumbed to disease. At 12 hours p.c. there were no bacteria associated with any tissue identified using IHC. By 36 hours p.c. moderate levels of bacteria were associated with lesions in the mesenteric lymph models of one animals, and a minimal level in the spleen. No bacteria where identified in animals by IHC at 48 hours p.c. and at 54 hours p.c. there was again substantial amount of bacteria in the mesenteric lymph node of one animal. Despite the high concentration of bacteria determined by plate culture in the gastrointestinal tract of animals that succumbed to disease, IHC only identified bacteria in the mesenteric lymph nodes of two animals and the duodenum of a further animal. This may be related to the sensitive or localisation of the IHC process or indicate that the bacteria obtained by plate culture may have been in the lumen and not in the tissue.

**Table 2:** Summary of the presence of *B*. *pseudomallei* antigen in various marmoset tissues following ingested challenge.

Animal ID	12 hour		36 hour		48 hour		54 hour		Terminal		
Stain	H&E	Bps	H&E	Bps	H&E	Bps	H&E	Bps	H&E	Bps	cfu/g
**Liver**	**-**	**-**	**(1/4)**	**-**	**+/- or + (2/4)**	**-**	**-**	**-**	**++ (2/4)**	-	2.0 x 10^7^ ± 7.0 x 10^6^
**Spleen**	**-**	**-**	**(1/4)**	**+/- (1/4)**	**+/- (2/4)**	**-**	**-**	**-**	**++ (2/4)**	-	6.5 x 10^7^ ± 5.9 x 10^7^
**Kidney**	**-**	**-**	**-**	**-**	**-**	**-**	**-**	**-**	**-**	**+/- (2/4)**	2.3 x 10^5^ ± 8.3 x 10^4^
**Lung**	**-**	**-**	**-**	**-**	**-**	**-**	**-**	**-**	**-**	**+/++ (2/4)**	1.5 x 10^6^ ± 7.6 x 10^5^
**Adrenal Gland**	**-**	**-**	**-**	**-**	**-**	**-**	**-**	**-**	**-**	**-**	N/D
**Inguinal LN**	**-**	**-**	**-**	**-**	**-**	**-**	**-**	**-**	**N/A**	**-**	N/D
**Oesophagus**	**-**	**-**	**-**	**-**	**-**	**-**	**-**	**-**	**-**	**-**	N/D
**Parotid**	**-**	**-**	**-**	**-**	**-**	**-**	**-**	**-**	**-**	**-**	N/D
**Stomach**	**-**	**-**	**-**	**-**	**-**	**-**	**-**	**-**	**-**	**-**	1.2 x 10^6^ ± 1.0 x 10^4^
**Duodenum**	**-**	**-**	**-**	**-**	**-**	**-**	**-**	**-**	**++ (1/4)**	**++ (1/4)**	2.9 x 10^7^ ± 2.0 x 10^7^
**Pancreas**	**-**	**-**	**-**	**-**	**-**	**-**	**-**	**-**	**-**	**-**	N/D
**Jejunum**	**-**	**-**	**-**	**-**	**-**	**-**	**-**	**-**	**++ (1/4)**	**-**	8.9 x 10^6^ ± 3.3 x 10^6^
**Ileum**	**-**	**-**	**-**	**-**	**-**	**-**	**-**	**-**	**-**	**-**	1.2 x 10^5^ ± 7.4 x 10^4^
**Caecum**	**-**	**-**	**-**	**-**	**-**	**-**	**-**	**-**	**-**	**-**	1.4 x 10^6^ ± 7.4 x 10^5^
**Colon**	**-**	**-**	**-**	**-**	**-**	**-**	**+ (1/4)**	**-**	**-**	**-**	7.0 x 10^5^ ± 3.2 x 10^5^
**Mesenteric LN**	**-**	**-**	**+/++(2/4)**	**++(1/4)**	**-**	**-**	**+/++(2/4)**	**+++(1/4)**	**++ (1/4)**	**+/+++ (2/4)**	N/D

- no lesions/immunological staining

+/- minimal lesions/immunological staining

+ mild lesions/immunological staining

++ moderate lesions/immunological staining

+++ severe lesions/immunological staining

N/D not determined

**Fig 3:**
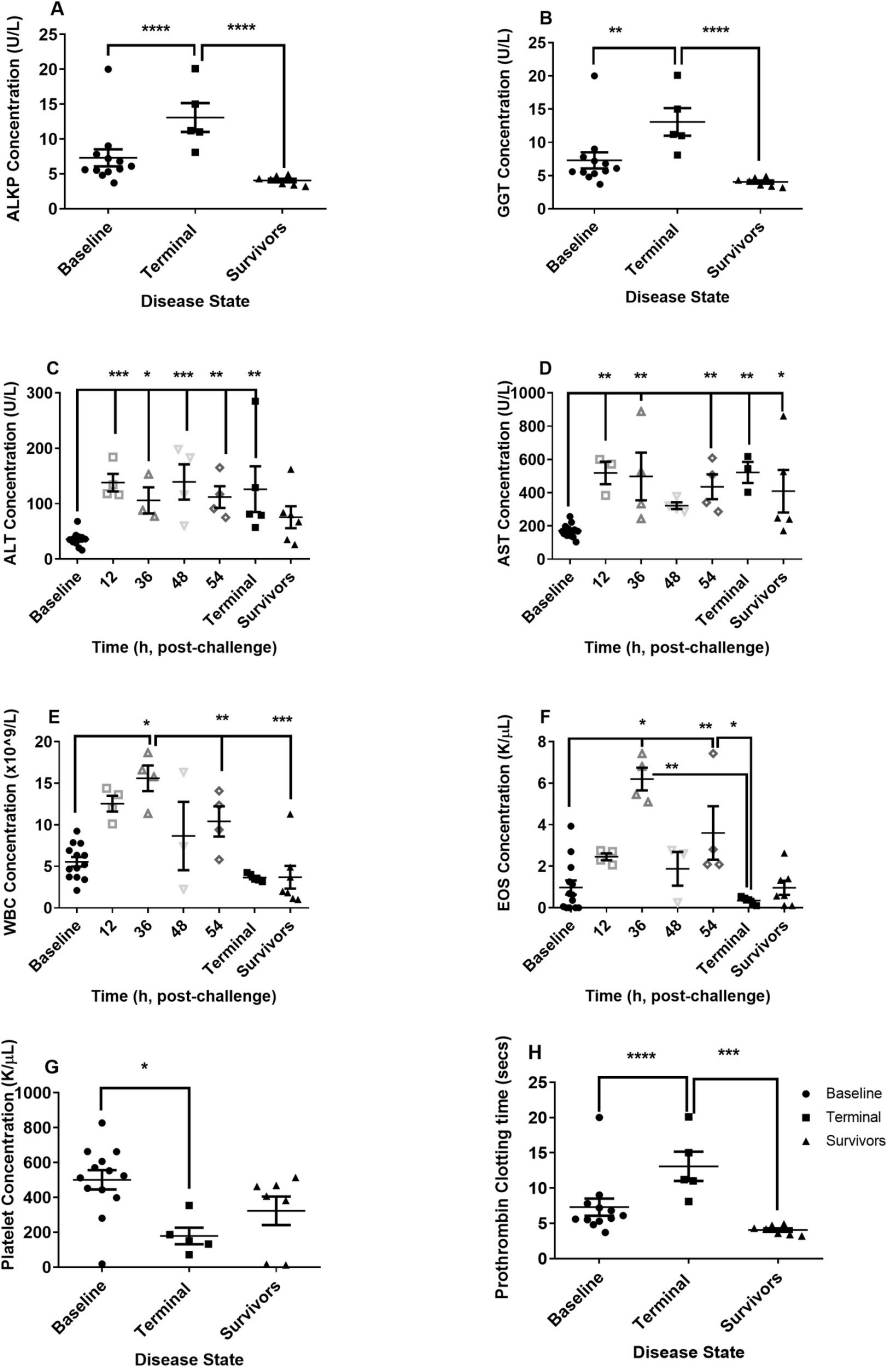
Clinical chemistry and haematological parameters observed in marmosets at the time of euthanasia following challenge with between 6.2 and 8.1 x 10^6^ cfu of *B*. *pseudomallei* by the ingested route. A ALKP (Alkaline phosphatase U/L) levels from the dose-ranging study, B GGT (Gamma-glutamyl Transferase U/L) levels from the dose-ranging study, C ALT (Alanine transaminase U/L), D AST (Aspartate aminotransferase U/L), E WBC (White Blood Cell Count x 10^9^/L), F EOS (Absolute eosinophil count, K/μL), G PLT (Platelets K/μL), H Prothrombin time (Secs) levels from the dose-ranging study. For C to G data from Study 1 (dose-ranging) and Study 2 (natural history) having been included for comparison purposes.

Haematological and clinical chemistry parameters in the blood were compared pre-challenge to blood collected at the time of post mortem. By 12 hours p.c. various clinical chemistry parameters were altered including elevation of levels of glucose, uric acid, and potassium levels and a decline in urea levels (S2 Fig). However, the most significant changes occurred in the liver enzymes (Fig 3A–3D). Two of the liver enzymes, ALKP and GGT only increased in animals that succumbed to infection. Two other liver enzymes, ALT and AST, steadily increased during disease (Fig 3C and 3D). Levels of both these enzymes were raised in surviving animals although only the increased levels of AST were significant. Generally, levels of potassium and creatinine continued to increase with time (S2 Fig). Elevated levels of creatinine were also apparent in animals that survived the disease. A range of other parameters, specifically bilirubin, amylase, creatine kinase, lactate dehydrogenase, sodium and the prothrombin time all altered significantly in terminal animals (Figs 3 and S2).

Few alterations were observed in the red blood cell (RBC) count; however changes in some of the RBC indices were apparent at 12 and 24 hours p.c. specifically an increase in the mean corpuscular volume (MCV) and a decrease in the mean cell haemoglobin concentration (MCHC). An increase in the levels of WBC and eosinophils were apparent at 36 hours p.c., which declined thereafter (Fig 3E and 3F). A lower level of lymphocytes was observed in animals that survived challenge (S3 Fig). An increase in the levels of monocytes was also apparent at 36 hours p.c., again declined thereafter. Thrombocytopenia was apparent in the animals that succumbed to disease as was alterations in the platelet indices including platelet haematocrit (PCT) (Figs 3G and S2).

### Immunological response to ingested *B*. *pseudomallei*

**Fig 4:**
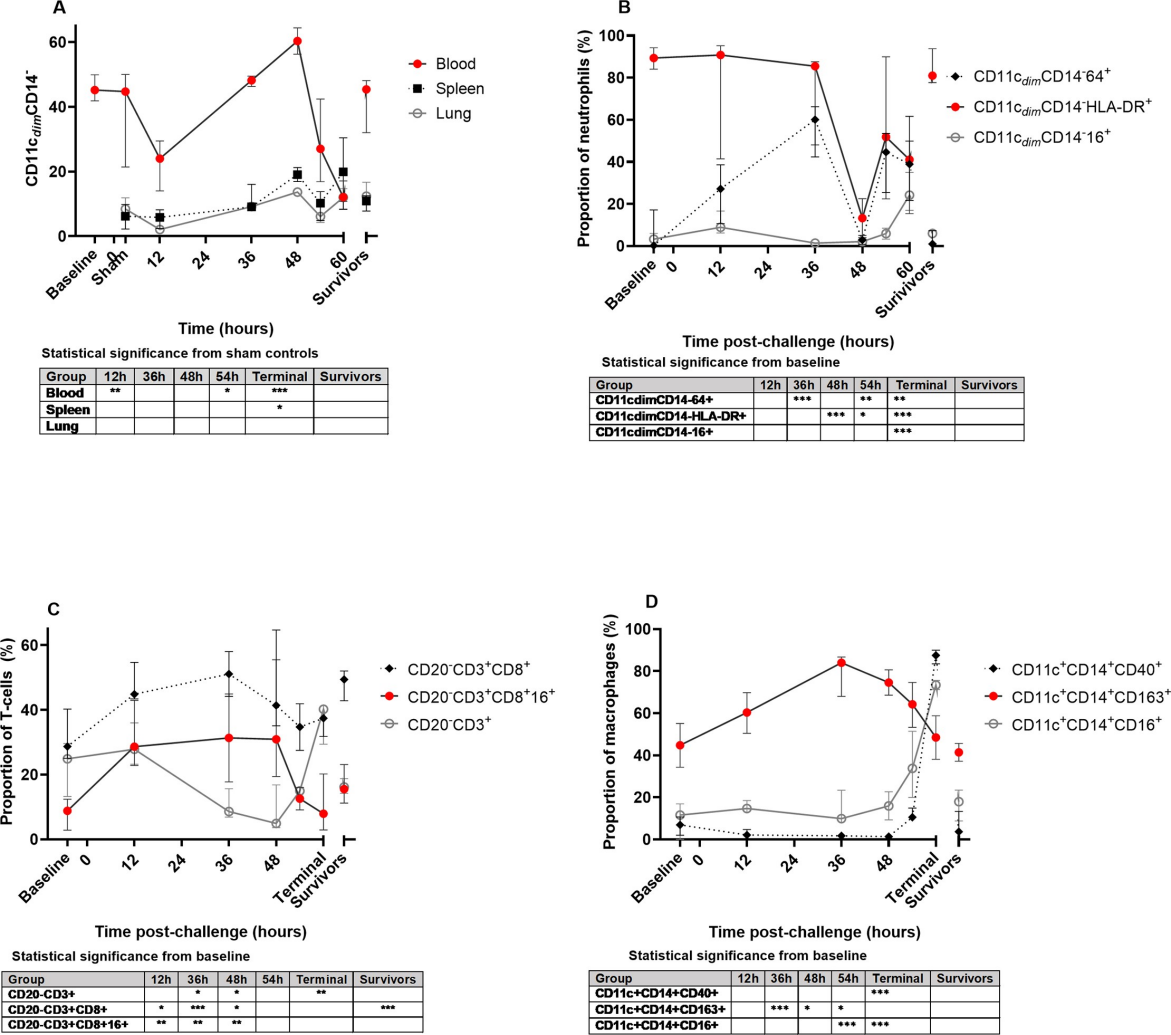
Phenotype and activation status in the blood, spleen and lungs of marmosets challenged with *B*. *pseudomallei* by the ingested route. A Proportion of neutrophils (CD11c_*dim*_ CD14^-^) in the blood, spleen and lungs, B Expression markers observed on neutrophils in the blood, C T-cells in the blood, D Macrophages in the blood.

The proportion of different immune cell types was assessed in the blood (pre and post challenge), and lung and spleen homogenates post-challenge (Fig 4). The most striking change is in the proportion of neutrophils from the blood in response to disease (Fig 4A). There is a significant decrease at 12 hours p.c. followed by an increase peaking at 48 hours, before significantly declining in animals that reached the humane endpoint. The same trend was observed in the tissues, until the humane end point was reached (Fig 4A).

Coinciding with the increase in neutrophil proportions in the blood, were increases in the expression of the distress/sepsis marker CD64^+^ and reductions in the maturation and functional markers CD16^+^ and HLA-DR (MHC II) on the neutrophils (Fig 4B). CD64^+^ expression also increased on neutrophils from the lungs until 48 hours p.c. but not on those from the spleen (S4 Fig), suggesting greater involvement of the lung than the spleen during disease. Neutrophil HLA-DR expression was significantly reduced in the blood, but also on neutrophils in the lung and spleen homogenates (all significant by 48 hours P>0.001). Other neutrophil activation markers (CD54^+^ and CD66b^+^) were less closely linked to the disease progression, but all activation/maturation markers (CD54^+^, HLA-DR, CD66b^+^ and CD16^+^) were reduced at 48 hours p.c. when neutrophils were most numerous in the blood. In animals surviving disease both the proportions of neutrophils and their activation/maturation markers were equivalent to baseline levels (pre-challenge), whereas for animals succumbing to disease these markers were significantly different.

There was a transient increase in lymphocytes in the blood at 12 hours p.c. (significant for B cells P>0.01) followed by a decrease by 48 hours p.c for both T and B cells by 54 hours p.c. Despite the general decline in blood T cells, the levels of CD8^+^ T cells and activated CD16^+^ CD8^+^ T cells increased during the course of disease (Fig 4C). A more subtle change in activation expression in the T cells from spleen and lung was observed, showing a general decline in the spleen and a general increase in the lungs. Animals that survived challenge had a significant increase in the proportion of circulating CD8^+^ T cells with a higher expression of activation markers than baseline.

There was a transient increase in monocytes/macrophages in the blood and lungs at 12 hours p.c. (P>0.05) but again no changes in the spleen. There was a reduction in HLA-DR expression on all macrophages from 48 hours p.c. in blood and spleen, an indication of sepsis. CD16^+^ (an indeterminate activation marker) increased on blood monocytes as disease progressed (Fig 4D) and was also significantly elevated in all lung samples (P>0.001). CD40^+^ (classical activation marker) also increased as disease progressed and was highly expressed on all macrophages in all samples from animals succumbing to disease (Fig 4D). CD163^+^ (an alternative activation marker), significantly increased up to 36 hours p.c, but then declined as CD40^+^ expression increased. As with neutrophils, macrophages/ monocytes were not significantly different between baseline levels and surviving animals.

Error bars are median and interquartile range, significance compared pre-challenge (Baseline) values or sham controls (Sham) to animals that succumbed (Terminal) or surviving animals (Survivors), significance is P>0.05 *, P>0.01** and P>0.001*** by one-way ANOVA. All data is from Study 2, natural history study, except for the “Terminal” timepoint where data from animal’s challenged with either 6.2 or 6.8 x 10^6^ cfu of *B*. *pseudomallei* has been included for comparison.

**Fig 5:**
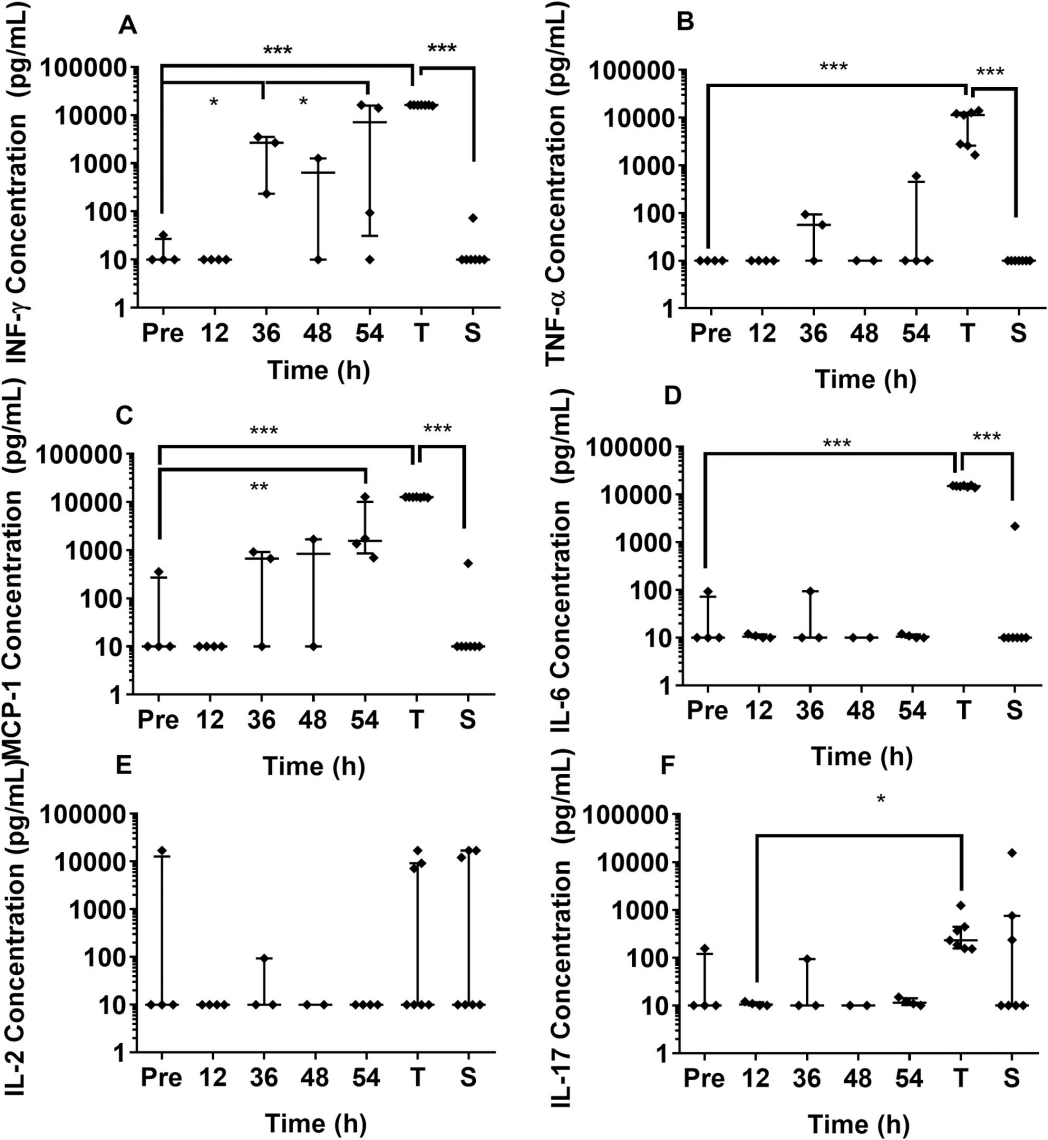
Levels of cytokines detected in blood from marmosets infected with *B*. *pseudomallei* by the oral route. A IFN-γ, B TNF-α, C MCP-1β (CCL4), D IL-6, E IL-2 F IL-17. Bars are median and interquartile range. **Pre** denotes pre-challenge blood samples, **T** is terminal disease (i.e. dose-ranging studies that succumbed to disease), **S** is survivors (i.e. dose-ranging studies that survived challenge). Statistical significance was determined by one-way ANOVA and is marked at the first time the cytokine level is significant compared to baseline (Pre) with P>0.05 *, P>0.01** and P>0.001***. All data is from Study 2, natural history study, except for the “Terminal” timepoint where data from animal’s challenged with between 6.2 and 6.8 x 10^6^ cfu of *B*. *pseudomallei* has been included for comparison.

The level of cytokines present in the blood of animals collected pre-challenge, and in the majority of animals that survived challenge, was below the detection limit of 10 pg/mL. IFN-γ was the first cytokine to be statistically significantly elevated at 36 hours p.c. with *B*. *pseudomallei* (Fig 5A). Levels of IFN-γ, TNF-α and MCP-1β increased with time until the animal succumbed to disease. However, levels of IL-6, IL-17 and IL-2 generally remained low until the animal succumbed. The concentration of IL-2 was variable in lethal disease with only 3 out of the 7 animals that succumbed having high levels of IL-2, but this was not statistically significant. This was also observed in animals that survived challenge.

### Lymphatic dissemination of bacteria from the intestine following infection

**Fig 6:**
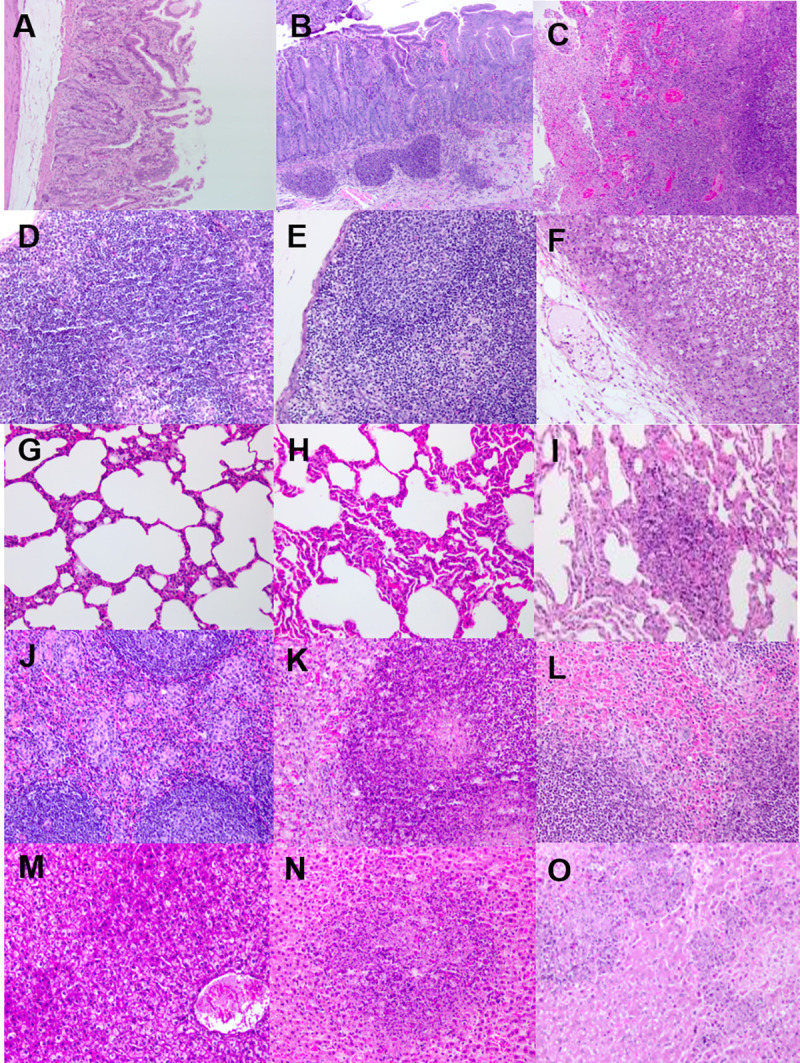
Representative tissues sections from marmosets that succumbed following challenge with *B*. *pseudomallei* by the ingested route. A H&E section from the jejunum of an naïve animal showing no lesions, B H&E section from the jejunum of an infected animal showing moderate lesions, C H&E section from the jejunum of an infected animal showing severe lesions, D H&E section from the mesenteric lymph node of an naïve animal showing no lesions, E H&E section from the mesenteric lymph node of an infected animal showing moderate lesions, F H&E section from the mesenteric lymph node of an infected animal showing severe lesions, G H&E section from the lungs of an naïve animal showing no lesions, H H&E section from the lungs of an infected animal showing moderate lesions, I H&E section from the lungs of an infected animal showing severe lesions, J H&E section from the spleen of an naïve animal showing no lesions, K H&E section from the spleen of an infected animal showing moderate lesions, L H&E section from the spleen of an infected animal showing severe lesions, M H&E section from the liver of an naïve animal showing no lesions, N H&E section from the liver of an infected animal showing moderate lesions, O H&E section from the liver of an infected animal showing severe lesions.

Enteritis (duodenitis, ileitis and/or jejunitis) was observed in sections of the small intestine from animals that succumbed to disease. The extension and severity of the lesions varied from mild to moderate lymphoplasmacytic and histiocytic infiltration in the lamina propria to focally extensive transmural acute necrotizing enteritis. A typical example of this was observed in the jejunum of one animal (Fig 6). This presented as acute focally extensive necrotizing jejunitis with a neutrophilic infiltration in mucosa and submucosa and the more internal areas of the muscular layer. Occasional, severe lytic lesions were observed in superficial areas of the mucosa, which had focal loss of the enterocyte layer and fibrin exudation and haemorrhage, and also extending deeper across the mucosa, submucosa and the muscular layer and serosa, and with thrombosis and vessel wall necrosis of the blood vessels of the affected area. These inflammatory lesions were most frequently found in areas of gut associated lymphoid tissue, obliterating the normal structure of the lymphoid tissue, and overlaying and surrounding tissues. The necrotic area extends between intact epithelium of crypts and villi with necrosis and fibrin deposition and thrombosis of the wall of the blood vessels of the affected areas. Small areas of necrosis were also evident in nearby areas of the submucosa, but separated by healthy tissue of the lesioned areas, suggesting a lymphatic dissemination. In colon, mild to moderate lymphoplasmacytic and histiocytic infiltration in lamina propria and the presence of gut associated lymphoid tissue (GALT) were observed in the colon of all animals that succumbed to disease, and occasionally in animals that survived challenge.

However, the most severe pathological features were observed in the mesenteric lymph nodes from animals that succumbed to disease. Multiple lymph nodes were examined from each animal and there was a variable severity and extension of acute neutrophilic and necrotizing lymphadenitis and perilymphadenitis (Fig 6). This ranged from small focal lesions of neutrophilic in infiltration in subcapsular location, to extensive diffuse necrotizing lesions affecting the whole cut surface. There were also necrotizing lesions in the mesenteric fat consistent with necrotizing lymphangitis and steatitis with focal haemorrhages in the surrounding mesenterium. Short rods, indicative of *B*. *pseudomallei*, were visible mixed with the necrotic material. These findings, including the pathological changes in the small intestine, are consistent with lymphatic draining as route of dissemination.

Dissemination then occurred to the spleen and liver, possibly haematogenous, where typical melioidosis disease-related pathological findings were observed; random multifocal necrotizing hepatitis or splenitis (Fig 6). Hepatitis ranged from moderate to severe in severity with evidence of the areas of necrosis extending into vascular structures resulting in necrotizing vasculitis, fibrin deposition and thrombosis. Despite the high levels of bacterial colonisation, negligible histopathological features were observed in the lungs of animals; however one animal had severe acute multifocal necrotizing interstitial pneumonia, the location of the lesion in septa also suggesting vascular dissemination (Fig 6I). The parotid gland, pancreas, kidney and stomach of these animals had no disease related features.

### Description of lesions

**Fig 7:**
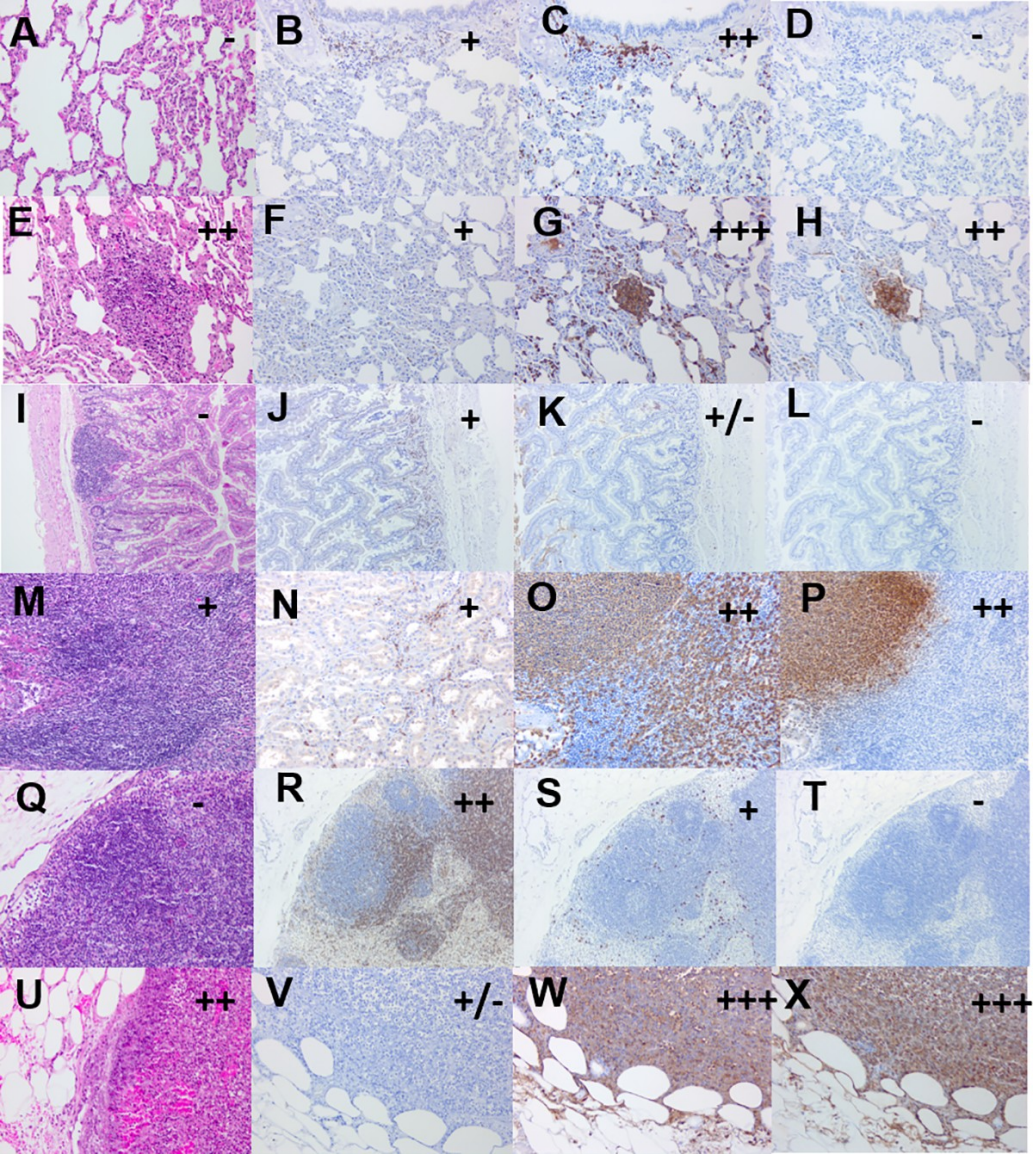
Representative tissues sections from marmosets that succumbed following challenge with *B*. *pseudomallei* by the ingested route. A H&E section from the lung of an infected animal showing no lesions at the humane endpoint, B CD3 stained section of the early lung lesion showing mild T-cells staining, C MAC387 stained section of the early lung lesion showing minimal macrophages/neutrophils staining, D stained section of the early lung lesion showing no bacterial antigen staining, E H&E section from the lung of an infected animal showing late lesions at the humane endpoint, F CD3 stained section of the late lung lesion showing mild T-cells staining, G MAC387 stained section of the late lung lesion showing severe macrophages/neutrophils staining, H stained section of the late lung lesion showing moderate bacterial antigen staining, I H&E section from the jejunum of an infected animal showing no lesions at 36 hours p.c., J CD3 stained section of the early jejunum lesion showing mild T-cells staining, K MAC387 stained section of the early jejunum lesion showing minimal macrophages/neutrophils staining, L stained section of the early jejunum lesion showing no bacterial antigen staining, M H&E section from the jejunum of an infected animal showing late lesions at 36 hours p.c., N CD3 stained section of the late jejunum lesion showing mild T-cells staining, O MAC387 stained section of the late jejunum lesion showing moderate macrophages/neutrophils staining, P stained section of the late jejunum lesion showing moderate bacterial antigen staining, Q H&E section from the mesenteric lymph node of an infected animal showing no lesions at 12 hours p.c., R CD3 stained section of the early mesenteric lymph node lesion showing moderate T-cells staining, S MAC387 stained section of the early mesenteric lymph node lesion showing mild macrophages/neutrophils staining, T stained section of the early mesenteric lymph node lesion showing no bacterial antigen staining, U H&E section from the mesenteric lymph node of an infected animal showing a moderate late lesions, V CD3 stained section of the late mesenteric lymph node lesion showing minimal T-cells staining, W MAC387 stained section of the late mesenteric lymph node lesion showing severe macrophages/neutrophils staining, X stained section of the late mesenteric lymph node lesion showing severe bacterial antigen staining. All images are x20 except E & I which are x10.

Three antibodies were used to further describe the lesions and surrounding areas from selected animals. The CD3 and MAC387 antibody markers were used to identify T-cells and macrophages/neutrophils (generic myeloid marker), respectively. The 3VE15 Bps antibody was used to detect *B*. *pseudomallei* capsular antigen. Generally, “early” lesions were associated with an influx of T-cells (CD3 labelled cells), neutrophils and macrophages (MAC387 labelled cells) to a potential infection site, which was not always associated with a visible lesion using H&E staining (Fig 7). Advanced lesions were associated with high numbers of neutrophils/macrophages and bacterial capsular antigen. In the absence of identifiable lesions by H&E staining in the kidney and lungs of all animals, there was minimal to mild staining with the CD3 and MAC387 antibodies (S5 Fig). However, when a lesion was identified it was associated with an increase in the MAC387 and Bps staining to severe and moderate levels, respectively. For the remaining tissues, in the absence of an identifiable H&E lesion, minimal to mild staining of CD3 and MAC387 was also observed. However, this was not associated with any staining of bacterial antigen.

## Discussion

The aim of this work was to develop a marmoset model of ingested melioidosis for comparison of the disease with other routes of infection. Ultimately these models will then be used to assess the efficacy of medical countermeasures.

Comparable to the inhalational and subcutaneous routes of *B*. *pseudomallei* infection in the marmosets, there was a significant dose-related time to the humane endpoint in the ingested model. Marmosets challenged with greater than 6.2 x 10^6^ cfu consistently succumbed to lethal disease. Lethal disease resulted in an acute, febrile response with all animals succumbing by 82 hours p.c. This latter time represents the humane endpoint for animals challenged with the lowest lethal challenge dose of 6.2 x 10^5^ cfu. It is difficult to compare lethality directly with the other routes of infection as the high challenge doses for ingestion were not routinely explored in the other marmoset models. However, animals that succumb to disease following subcutaneous and inhalational infection around 82 hours p.c. had a challenge dose of 1.2 x 10^2^ and 4 cfu of bacteria respectively. This observation suggests that the inhalational route of challenge is more lethal than the subcutaneous route (approximately 2 logs more bacteria required to cause lethal disease in a similar timeframe). The subcutaneous route is in turn more lethal that the ingested route that requires 4 logs more bacteria to cause lethal disease in a similar timeframe.

Death in humans due to melioidosis has been historically reported to occur between 24 and 48 hours following severe symptoms [22]. In the marmoset ingestion model, animals typically reach the humane endpoint from 22 to 41 hours after the onset of fever. Mortality in humans varies between Australia and Thailand from between 10% to 50%, respectively, reflecting different routes of infection, different strains, risk factors, and clinical practices [23]. At lower challenges in the marmoset the mortality rate was reduced to 25% at approximately 1 x 10^5^ cfu and 0% at less than 1.3 x10^4^ cfu for the duration of the study. The animals that survived to the end of the study had evidence of a subclinical disease including raised cytokine levels and liver enzymes. However, no viable bacteria where recovered from any tissue assessed and there was no evidence of any microscopic pathological features. These animals may have developed a chronic infection with time, which may or may not have resulted in lethal disease. Lethal disease observed in marmosets in this study, and previously reported in mice require high challenge doses; 6 x 10^6^ in marmosets and 1 x 10^8^ cfu in mice [10]. However, drinking water in endemic regions that have been associated with ingested disease only contains between 1–65 cfu/L [24]. Clinically, most human cases associated with ingestion present as chronic or even subclinical disease with lethal disease being a rare event. Outbreaks of cases associated with water plant contamination etc, may be associated with high bacterial doses. Animal models with clear, measureable clinical readouts are advantageous for assessing medical countermeasures as protective efficacy is easy to determine.

Animals that succumbed to lethal disease showed the typical features of melioidosis that had previously been observed in marmosets following challenge by other routes [17,18]. These included changes in haematological and clinical chemistry parameters that are associated with human melioidosis such as increased white blood cell counts, liver function enzymes, creatinine and urea, thrombocytopenia and high activation of the coagulation system [25,26,27]. Following challenge by the ingested route there was only evidence of a significantly raised PT in animals that had reached the humane endpoint, and a slight increase in the aPPT time in some animals during the disease progression. Following inhalational challenge in marmosets a prolonged aPPT was observed from 12 hours p.c. PT and aPPT are indicators of the extrinsic and intrinsic coagulation pathway, respectively, and the difference in the clotting factor between routes of infection may be indicative of a different cause of the coagulopathy, as well as reflecting the difference in the disease.

An early innate response is observed following challenge by the ingested route, with an initial decrease in the number of neutrophils in the blood as they migrate to the lung and spleen. Two early markers of disease were observed based on the expression of HLA-DR and CD64^+^ on the neutrophils. CD64^+^ is an activation marker expressed on the surface of neutrophils during acute infections (or sepsis) of patients being treated in high dependency hospital units [28]. The expression of CD64^+^ on neutrophils combined with loss of HLA-DR expression on macrophages is being considered as a diagnostic marker of infection for hospital admissions [29]. This study has shown that elevation of CD64^+^ in blood is linked to disease, and is significantly increased before the animals show clinical signs.

One of the most surprising features of the ingested disease is the early colonisation of the lung, with between 1 x 10^3^ and 1 x 10^4^ cfu of bacteria observed in the lungs from 12 hours p.c. despite the absence of bacteraemia. It is unlikely that the bacteria entered the lungs due to aspiration during challenge. The bacteria were administered in 100 μL volumes in a 1 mL suspension of Nesquik which the animals readily took from a syringe. Animals were fully conscious and there was no evidence of “spluttering” which would have occurred if the animals had accidentally inhaled any of the bacteria during the drinking process. Inhalational delivery results in a rapid multiplication in the lungs which is not observed following ingestion as the number of bacteria remain relatively constant [17]. This suggests that the bacteria enter via a different mechanism. By 36 hours p.c. high concentrations of bacteria are then recovered from the spleen and liver, with no proliferation in the lungs. By the time the animal succumbed to disease, the highest concentrations of bacteria are found in the liver and spleen, followed by the lungs. This is different to both the subcutaneous and aerosol routes of infection. Following inhalational challenge bacteria multiply rapidly in the lungs and disseminate throughout the body, primarily the kidney and spleen by 24 hours p.c. When animal’s succumbed to disease, the highest bacterial concentrations remain in the lungs followed by the spleen and liver. Pathologically inhalational animals have necrotising pneumonia, splenitis and hepatitis. To date, only one animal has exhibited lung lesions following ingested disease. However, this was localised and suggestive of vascular dissemination. Following subcutaneous challenge the bacteria spread from the site of inoculation to the endoreticular system, initially the spleen followed by the liver. In animals that succumb to disease the highest concentration of bacteria are found in the liver and spleen followed by the kidney, lungs and blood. For both the inhalational and subcutaneous routes of exposure, bacterial spread is believed to be haematogenous. However, bacterial spread following ingestion may be through the lymphatic system. The bacteria may move from the gastrointestinal tract to the lymphatic system by bacterial translocation via the mesenteric lymph node. This is supported by the most severe microscopic inflammation occurring in the mesenteric lymph node. Bacteria would then exit the lymphatic system at the thoracic duct into the left jugular and subclavian vein that join the superior vena cava on the way to the right atrium of the heart. The blood containing the bacteria would then get pumped to the lung for oxygenation which would explain the early recovery of bacteria in the lungs. From the lungs, the blood and bacteria would then get pumped around the body, reaching the spleen were the bacterial numbers proliferate. The high incidence of systemic and pulmonary diseases following gut injury is hypothesised to be due to bacterial translocation [30]. Similarly gut bacteria can be cultured in the mesenteric lymph nodes and may cause opportunistic infections using the mechanism described above [31]. In marmoset ingestional melioidosis, the numbers of bacteria in the lungs remain at a constant level throughout the disease with a small increase in animals that succumb to disease. Contrastingly, in inhalational disease, the bacterial numbers continue to increase in the lungs throughout the disease associated with an increase in the severity of pneumonia.

Surprisingly, there was no evidence of parotitis observed in the marmosets, despite this having a clear link with ingestional disease, particularly in children in Thailand [32]. Parotitis may be a feature of a more chronic form of the disease, while the marmoset model focused on acute disease. It would have been intriguing to determine whether chronic disease would have manifested in animals that survived the initial challenge without any clinical disease.

Additionally of note is the increased level of eosinophils during the course of *B*. *pseudomallei* infection by the oral route in comparison to melioidosis by other routes. Absolute levels of eosinophils peak at 14 K/μL at 36 hours p.c. following the ingestion, with levels typically at 1–2 K/μL at 36 hours p.c following challenge by the inhalation or subcutaneous routes. This may be an important biomarker to determine the causative route of infection. Eosinophils are not routinely involved in the host response to bacterial infection, indeed they are typically decreased in bacterial disease [33]. However, they do play an important role in the host response to parasites as well as been linked to inflammatory bowel disease. Typically, eosinophils are abundant in the lamina propria of the gastrointestinal tract, typically 10-fold higher numbers than in circulation [34]. Stimulation of the gastrointestinal tract following ingestion of *B*. *pseudomallei* may result in the eosinophils entering the circulatory system. This could be either via the blood supply to the liver or via the lymphatic system similarly to the proposed route of *B*. *pseudomallei* entry discussed above.

## Conclusions

*B*. *pseudomallei* is consistently lethal following ingestion of greater than 6.2 x 10^6^ cfu with all animals becoming febrile and succumbing to disease between 60 to 81 hours p.c. in a dose-related manner. The lung was the first organ to become colonised directly from the gastrointestinal tract and the most severe, consistent pathological features was observed in the mesenteric lymph nodes with severe multifocal to diffuse necrotizing lymphadenitis and perilymphadenitis. Marmoset models of melioidosis, with clear, measureable clinical readouts, are now available to compare the efficacy of antibiotics against melioidosis resulting from the three acquisition routes of the disease, inhalation, cutaneous and ingestion.

## Supporting information

S1 Table Criteria used to Determine Cell Phenotype and Activation Status. (DOCX)

S1 Fig Bacterial load in marmoset tissue at post-mortem following challenge with *B*. *pseudomallei* by the ingestion route. A Kidney, B Blood, C Liver. All data is from Study 2, natural history study, except for the “Terminal” timepoint where data from animal’s challenged with between 6.2 and 6.8 x 10^6^ cfu of *B*. *pseudomallei* has been included for comparison.
(TIF)

S2 Fig Clinical chemistry and haematological parameters observed in marmosets at the time of euthanasia following challenge with between 6.2 and 8.1 x 10^6^ cfu of *B*. *pseudomallei* by the ingested route. A K (potassium), B CREA (creatinine), C TBIL (total bilirubin), D AMYL (amylase), E CK (creatinine kinase), F LDH (lactate dehydrogenase), G Na (sodium), H PCT (platelet hematocrit), I GLU (glucose), J URIC (uric acid). Data from Study 1 (dose-ranging) and Study 2 (natural history) having been included for comparison purposes.
(TIF)

S3 Fig White blood cell differential observed in marmosets at the time of euthanasia following challenge with between 6.2 and 8.1 x 10^6^ cfu of *B*. *pseudomallei* by the ingested route. A NEU (neutrophils), B LYM (lymphocytes), C MONO (monocytes), D EOS (eosinophils), E BASO (basophils).
(TIF)

S4 Fig Activation status of neutrophils in the spleen and lungs of marmosets challenged with *B*. *pseudomallei* by the ingested route. A Proportion of neutrophils expressing HLA-DR) B Proportion of neutrophils expressing CD64^+^. Error bars are median and interquartile range, significance compared pre-challenge (Baseline) values or sham controls (Sham) to animals that succumbed (Terminal) or surviving animals (Survivors). All data is from Study 2, natural history study, except for the “Terminal” timepoint where data from animal’s challenged with either 6.2 or 6.8 x 10^6^ cfu of *B*. *pseudomallei* has been included for comparison.
(TIF)

S5 Fig Representative tissues sections from marmosets that succumbed following challenge with *B*. *pseudomallei* by the ingested route. A H&E section from the kidney, B CD3 stained section of the Kidney, C MAC387 stained section of the kidney, D H&E section from the lung, E CD3 stained section of the lung, F MAC387 stained section of the lung.
(TIF)
