# Supplementary material for: The lymphatic system as a potential mechanism of spread of melioidosis following ingestion of Burkholderia pseudomallei
Source: PLoS Negl Trop Dis. 2021 Feb 22;15(2):e0009016. doi: 10.1371/journal.pntd.0009016 (PMC7932547; doi:10.1371/journal.pntd.0009016)
Supplement: S1 Table — (DOCX) [file pntd.0009016.s001.docx]

**S1 Table. Criteria used to Determine Cell Phenotype and Activation Status.**

| **Cell Type** | **Identification markers** |
| --- | --- |
| B cells (as percentage of total cells) | CD20+ CD3- |
| Immature B cells (as percentage of B cells) | CD20+ CD3- CD16+ |
| NK cells (as percentage of total cells) | CD20- CD3- CD56+ |
| Mature NK cells (as percentage of NK cells) | CD20- CD3- CD56+ CD16+ |
| Activated NK (as percentage of NK cells) | CD20- CD3- CD56+ CD69+ |
| T cells (as percentage of total cells) | CD20- CD3+ |
| Cytotoxic T cells (as percentage of T cells) | CD20- CD3+ CD56+ CD16- |
| CD8 T cells (as percentage of T cells) | CD20- CD3+ CD8+ |
| NKT Cells (as percentage of CD8s) | CD20- CD3+ CD8+ CD56+ |
| True cytotoxic CD8s (as percentage of CD8s) | CD20- CD3+ CD8+ CD56+ CD16- |
| Early activated CD8 cells (as percentage of CD8s) | CD20- CD3+ CD8+ CD69+ |
| Activated CD8 cells (as percentage of CD8s) | CD20- CD3+ CD8+ CD16+ |
| CD4 T cells (as percentage of T cells) | CD20- CD3+ CD8- |
| NKT Cells (as percentage of CD4s) | CD20- CD3+ CD8- CD56+ |
| Early activated CD4 cells (as percentage of CD4s) | CD20- CD3+ CD8- CD69+ |
| Activated CD4 cells (as percentage of CD4s) | CD20- CD3+ CD8- CD16+ |
| Neutrophils (as percentage of total cells) | CD11c dim CD14- |
| Activated neutrophils (as percentage of Ns) | CD11c dim CD14- CD16+ |
| Adhesion positive neutrophils (percentage of Ns) | CD11c dim CD14- CD54+ |
| Antigen presenting neutrophils (percentage of Ns) | CD11c dim CD14- MHCII+ |
| Neutrophils expressing human activation marker (percentage of Ns) | CD11c dim Cd14- CD66b+ |
| Neutrophils expressing sepsis marker (percentage of Ns) | CD11c dim Cd14- CD64+ |
| Macrophages (as percentage of total cells) | CD11c + CD14+ |
| Classical M1 macrophages (as percentage of M0) | CD11c + CD14+ CD40+ |
| Alternative M2a macrophages (percentage of M0) | CD11c + CD14+ CD163+ |
| Intermediate macrophages (percentage of M0) | CD11c + CD14+ CD16+ |
| Inflammatory M1 macrophages (percentage of M0) | CD11c + CD14+ CD80+ |
| Super Antigen presenting (percentage of M0) | CD11c + CD14+ MHCII++ |
| Adhesion positive ( percentage of M0) | CD11c + CD14+ CD54+ |
| Immature macrophages (percentage of M0) | CD11c + CD14+ CD16- CD163- CD40- |
