# Supplementary figures and images for: The lymphatic system as a potential mechanism of spread of melioidosis following ingestion of Burkholderia pseudomallei
Source: PLoS Negl Trop Dis. 2021 Feb 22;15(2):e0009016. doi: 10.1371/journal.pntd.0009016 (PMC7932547; doi:10.1371/journal.pntd.0009016)

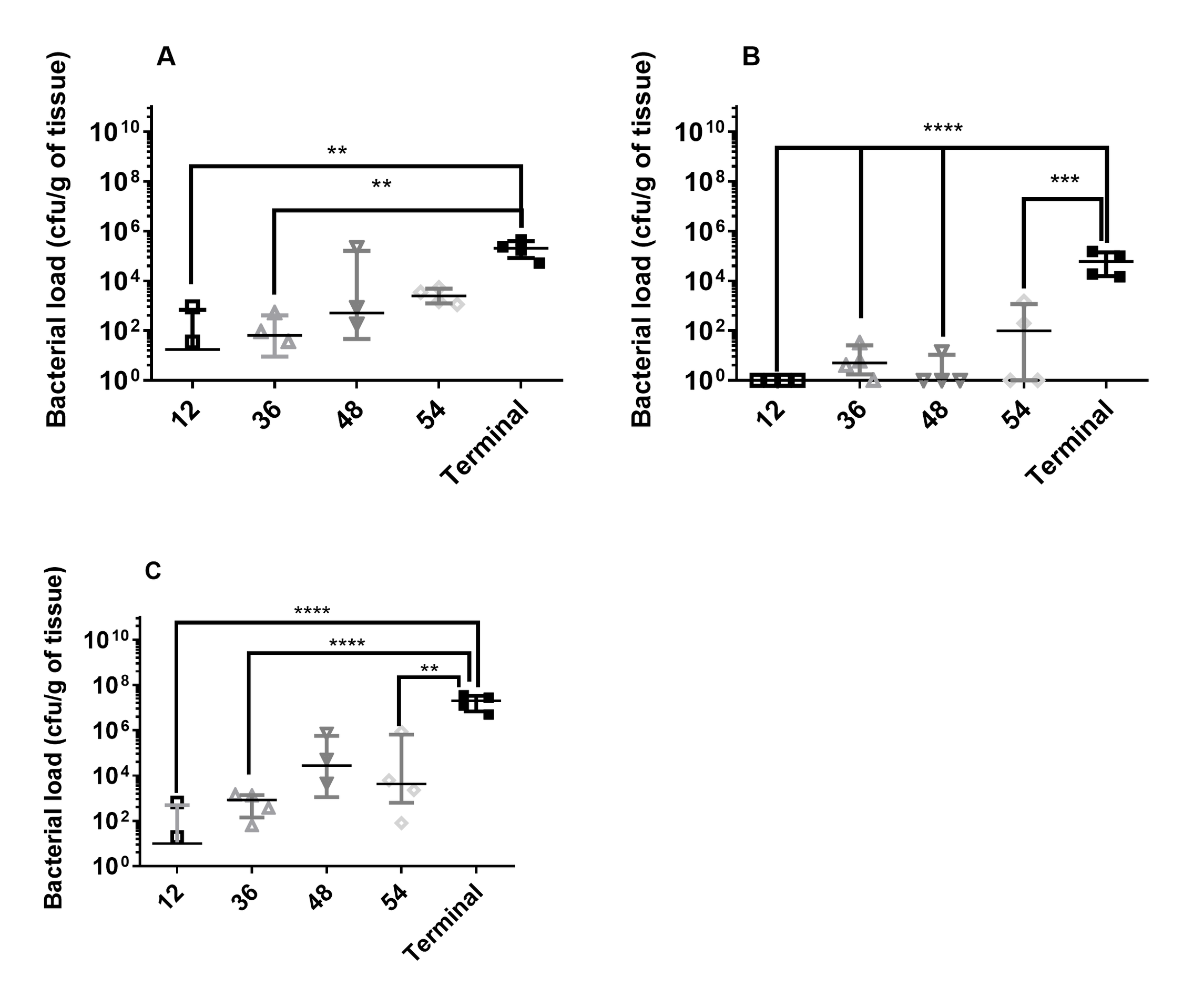

Supplement: S1 Fig — A Kidney, B Blood, C Liver. All data is from Study 2, natural history study, except for the “Terminal” timepoint where data from animal’s challenged with between 6.2 and 6.8 x 10^6^ cfu of B. pseudomallei has been included for comparison. (TIF) [file pntd.0009016.s002.tif]

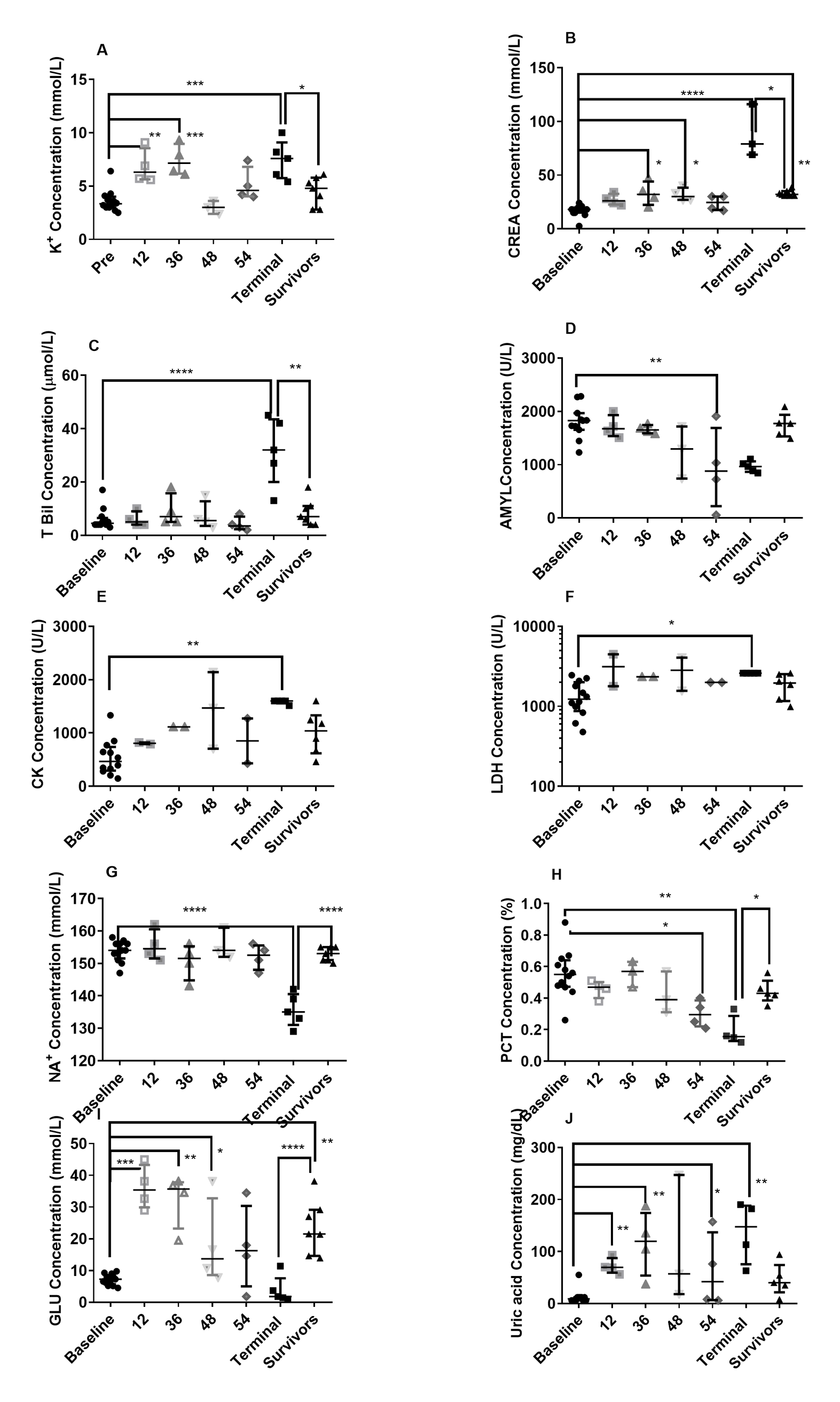

Supplement: S2 Fig — A K (potassium), B CREA (creatinine), C TBIL (total bilirubin), D AMYL (amylase), E CK (creatinine kinase), F LDH (lactate dehydrogenase), G Na (sodium), H PCT (platelet hematocrit), I GLU (glucose), J URIC (uric acid). Data from Study 1 (dose-ranging) and Study 2 (natural history) having been included for comparison purposes. (TIF) [file pntd.0009016.s003.tif]

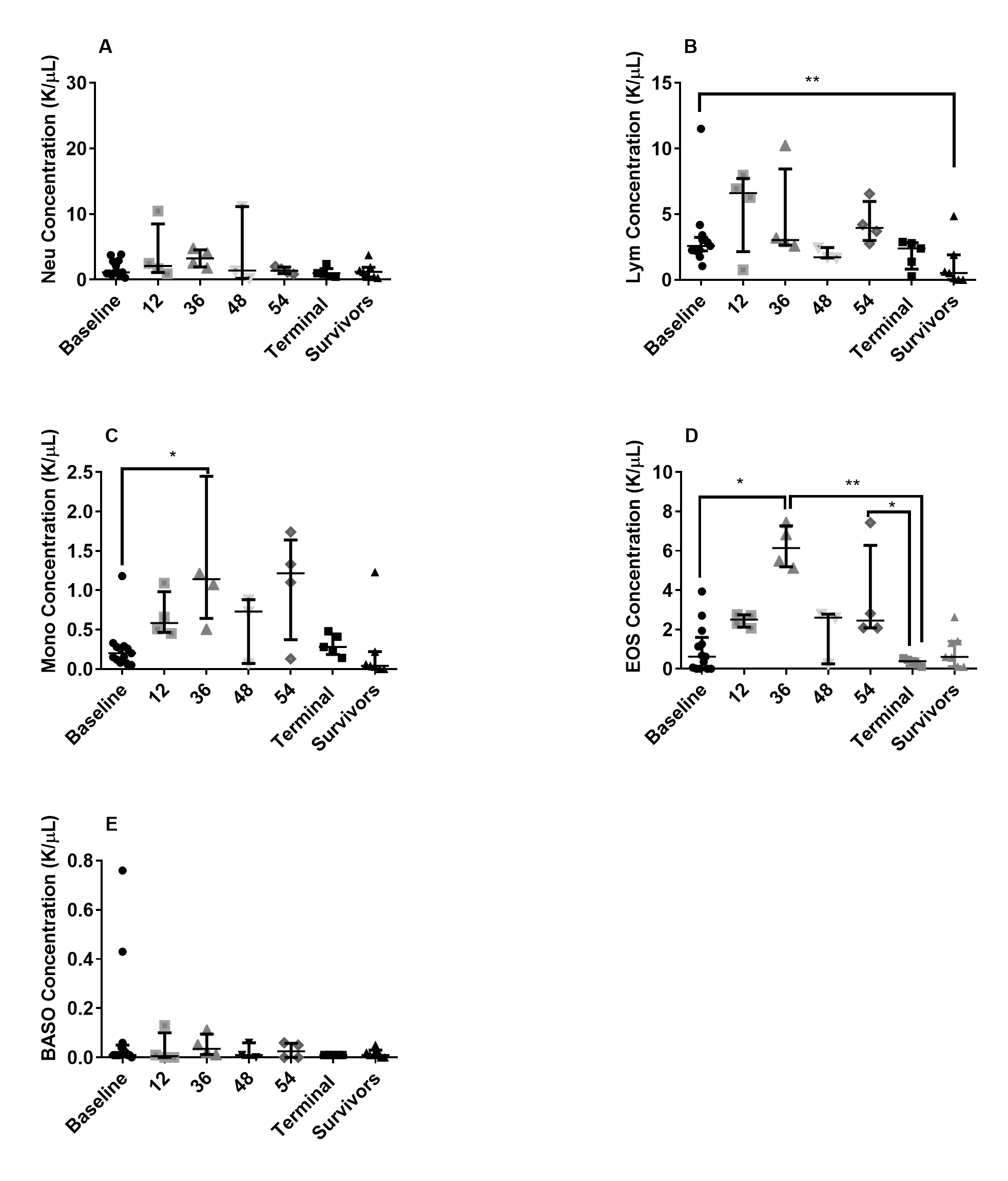

Supplement: S3 Fig — A NEU (neutrophils), B LYM (lymphocytes), C MONO (monocytes), D EOS (eosinophils), E BASO (basophils). (TIF) [file pntd.0009016.s004.tif]

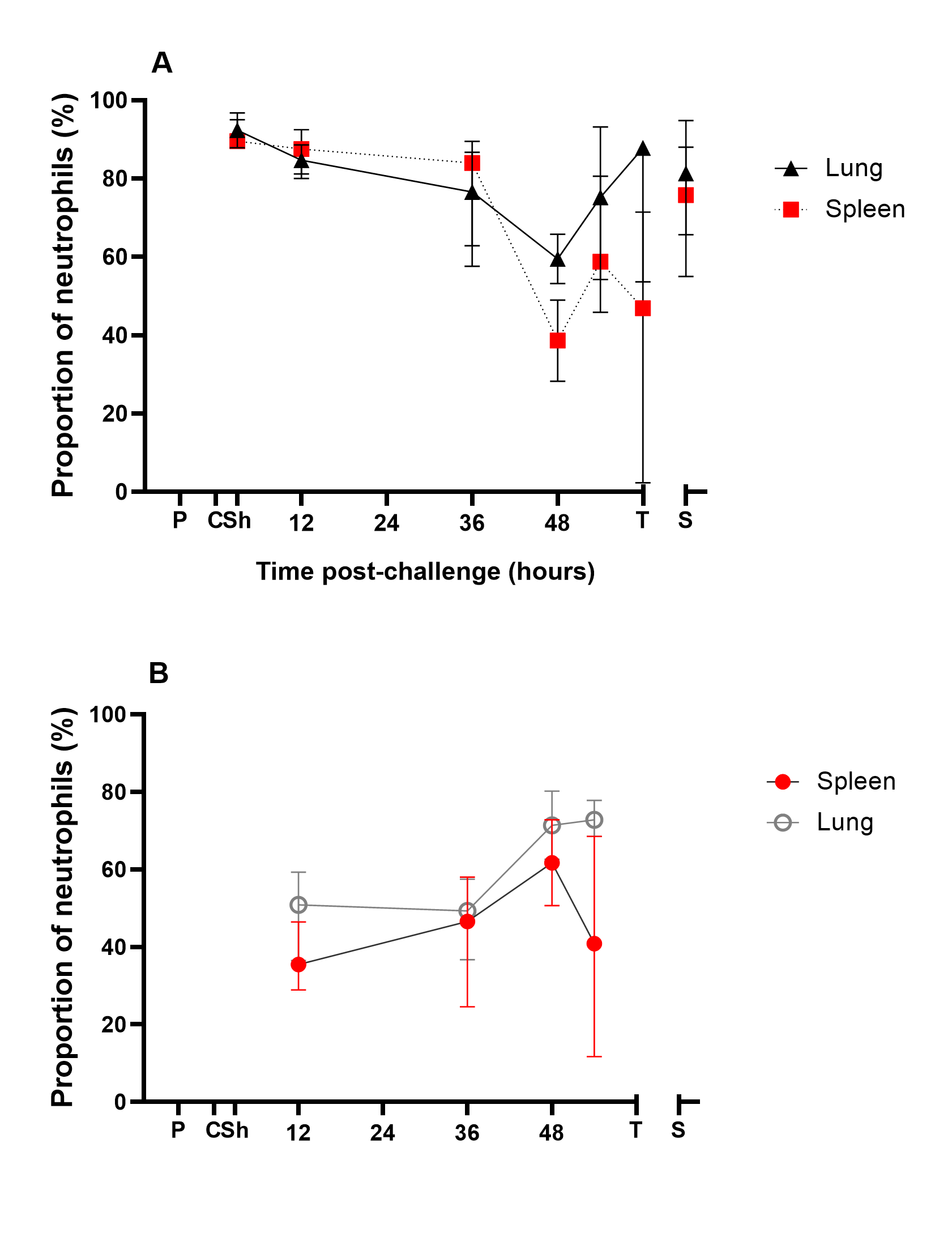

Supplement: S4 Fig — A Proportion of neutrophils expressing HLA-DR) B Proportion of neutrophils expressing CD64+. Error bars are median and interquartile range, significance compared pre-challenge (Baseline) values or sham controls (Sham) to animals that succumbed (Terminal) or surviving animals (Survivors). All data is from Study 2, natural history study, except for the “Terminal” timepoint where data from animal’s challenged with either 6.2 or 6.8 x 10^6^ cfu of B. pseudomallei has been included for comparison. (TIF) [file pntd.0009016.s005.tif]

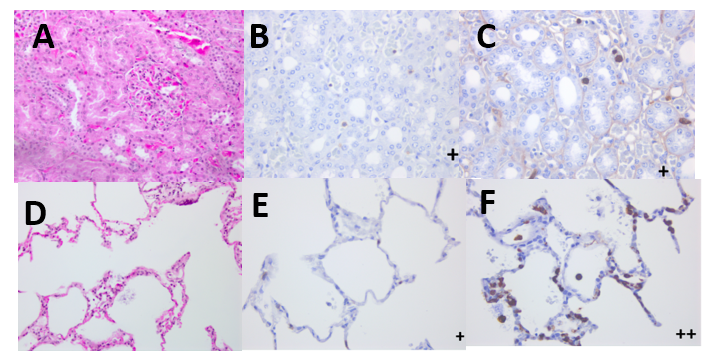

Supplement: S5 Fig — A H&E section from the kidney, B CD3 stained section of the Kidney, C MAC387 stained section of the kidney, D H&E section from the lung, E CD3 stained section of the lung, F MAC387 stained section of the lung. (TIF) [file pntd.0009016.s006.tif]
